# Multiple system atrophy: cure and care

**DOI:** 10.1007/s00702-026-03235-8

**Published:** 2026-07-25

**Authors:** Jolanda Buonocore, Bianca Caliò, Fabian Leys, Franziska Hopfner, Günter U. Höglinger, Andrea Quattrone, Nadia Stefanova, Alessandra Fanciulli

**Affiliations:** 1 Department of Neurology, Medical University of Innsbruck, Anichstraße 35, 6020 Innsbruck, Austria; 2 Neuroscience Research Center, University “Magna Graecia”, Catanzaro, Italy; 3 Department of Medical and Surgical Sciences, Institute of Neurology, University “Magna Graecia”, Catanzaro, Italy; 4 Department of Neurology, LMU University Hospital, LMU Munich, Munich, Germany; 5 German Center for Neurodegenerative Diseases (DZNE) Munich, Munich, Germany; 6 Munich Cluster for Systems Neurology (SyNergy), Munich, Germany; 7 Laboratory for Translational Neurodegeneration Research, Division of Neurobiology, Department of Neurology, Medical University of Innsbruck, Innsbruck, Austria

**Keywords:** Multiple system atrophy, α-synuclein, Glial cytoplasmic inclusions, Prodromal phase, Autonomic failure, Disease-modifying therapies, Multidisciplinary care

## Abstract

Multiple system atrophy (MSA) is a rare and rapidly progressive neurodegenerative disorder characterized by a variable combination of autonomic failure, parkinsonism, and cerebellar ataxia, with a median survival of 8–10 years from symptom onset. Its aetiology remains poorly understood, as most cases are sporadic and environmental contributors remain unclear. Neuropathologically, MSA is defined by the accumulation of α-synuclein within oligodendroglial cells, forming glial cytoplasmic inclusions that drive widespread neurodegeneration in striatonigral and olivopontocerebellar systems. Recent advances in diagnostic criteria and biomarker development have improved disease recognition; however, early diagnosis remains challenging, particularly during the prodromal phase, when clinical features overlap with other α-synucleinopathies. Advances in fluid biomarkers and multimodal imaging are expected to facilitate earlier detection, improve diagnostic accuracy, and provide more robust tools for monitoring disease progression. Therapeutic development has largely focused on targeting α-synuclein pathology, but these approaches have not yet demonstrated consistent clinical benefit, highlighting the biological complexity of MSA. In the absence of curative treatments, management remains largely supportive, aimed at symptom control, particularly addressing autonomic dysfunction and motor impairment. Emerging care models, including telemedicine and multidisciplinary management, are reshaping clinical practice and may improve access to specialized care. Bridging advances in pathophysiology with patient-centred care will be essential for improving outcomes in MSA.

## Introduction

Multiple system atrophy (MSA) is a rare and rapidly progressive neurodegenerative disorder characterized by a variable combination of autonomic failure, parkinsonism, and cerebellar ataxia (Krismer et al. 2024a). Clinically, it is classified into two main phenotypes based on the predominant motor features: the parkinsonian variant (MSA-P), characterized mainly by akinetic-rigid symptoms, and the cerebellar variant (MSA-C), in which gait and limb ataxia predominate.

The estimated incidence is approximately 0.6 per 100,000 person-years, with a prevalence ranging from 0.5 to 17 per 100,000, varying significantly by geographic region (Fanciulli and Wenning 2015). The disease carries a poor prognosis, with a median survival of 8–10 years from symptom onset (Wenning et al. 2013; Low et al. 2015).

In recent years, growing efforts have focused on elucidating the molecular and cellular mechanisms underlying MSA, alongside the development of biomarkers and refinement of diagnostic criteria. These advances have improved disease characterization and may facilitate earlier diagnosis, particularly during the prodromal phase. At the same time, emerging insights into α-synuclein biology have highlighted the need to distinguish physiological functions from the misfolding and aggregation processes that drive disease onset and progression, and suggest that MSA arises from multiple, converging mechanisms, including pathological α-synuclein aggregation, neuroinflammation, and loss of neurotrophic support (Sidoroff et al. 2022; Stefanova and Wenning 2023).

Despite these advances, major challenges remain, particularly in achieving early diagnosis and developing effective disease-modifying therapies.

This review summarizes recent advances in the understanding of MSA, focusing on genetic and pathophysiological mechanisms, evolving diagnostic frameworks, emerging biomarkers, and novel therapeutic strategies, with particular attention to their implications for clinical management and patient outcomes.

## Aetiology and putative genetic contributors

The aetiology of MSA remains poorly understood. Most cases occur sporadically, and consistent environmental risk factors have not been clearly identified (Fanciulli and Wenning 2015). Several case–control studies have investigated potential environmental contributors. Occupational exposure to organic solvents, pesticides, and heavy metals has been examined, with some reports suggesting an increased risk among agricultural workers and individuals employed in the plastics industry, but these associations have not been consistently replicated across populations (Hanna et al. 1999; Vidal et al. 2008). Supporting a possible role of occupational exposures, a recent analysis of work histories from 270 neuropathologically confirmed MSA cases in the Mayo Clinic Brain Bank identified an overrepresentation of several occupational sectors (i.e. petroleum industry or metal industry) compared with population data (Cheshire et al. 2025).

More broadly, growing evidence suggests that environmental factors may interact with genetic susceptibility in shaping disease risk, with reported associations involving alcohol consumption, well-water intake, pesticide exposure, and smoking (Kuo et al. 2022). An inverse association between cigarette smoking and MSA has been previously reported, consistent with observations in Parkinson’s disease (PD), although the link appears less pronounced (Vanacore 2005). This finding provides a rationale for investigating other environmental exposures implicated in PD but that remain underexplored in MSA (Krzyzanowski et al. 2025; Lin et al. 2026).

Despite the sporadic nature of MSA, the genetic contribution has also been increasingly investigated, with epidemiological studies suggesting a relatively modest heritability (approximately 2.09% and 6.65%) (Federoff et al. 2016). Nonetheless, rare multiplex families showing apparent autosomal dominant or recessive inheritance patterns have been reported (Wüllner et al. 2004; Hara et al. 2007; Fujioka et al. 2014).

Because the pathological hallmark of MSA is the accumulation of α-synuclein within glial cytoplasmic inclusions (GCIs), early genetic studies primarily focused on *SNCA*, the gene encoding this protein (Al-Chalabi et al. 2009; Scholz et al. 2009). Rare coding variants have not been consistently replicated across studies (Kiely et al. 2013; Pasanen et al. 2014), and associations with common single nucleotide polymorphisms have produced variable results depending on population and study design (Al-Chalabi et al. 2009; Scholz et al. 2009; Yun et al. 2010). Copy-number gains at the *SNCA* locus, however, provide indirect evidence that increased α-synuclein dosage may contribute to disease pathogenesis, including in clinical or pathological phenotypes suggestive of MSA (Fuchs et al. 2007; Mokretar et al. 2018; Garcia-Segura et al. 2023; Kon et al. 2024).

Beyond α-synuclein–related mechanisms, candidate-gene studies have explored additional biological pathways potentially involved in MSA pathogenesis. Variants in *COQ2*, implicated in mitochondrial function and oxidative stress pathways, were initially associated with MSA in Japanese families, with evidence supporting primarily autosomal recessive inheritance, although heterozygous variants have also been proposed as susceptibility factors (MSARC 2013). This association has not been replicated in European or North American populations (Sharma et al. 2014; Ogaki et al. 2014).

Research has therefore expanded to genes implicated in related neurodegenerative disorders. Among genes associated with PD, the involvement of *LRRK2* in MSA remains controversial. Although several studies have found limited evidence for a direct association with MSA risk (Ozelius et al. 2007; Heckman et al. 2014), other reports have suggested that *LRRK2* variants may influence disease phenotype and progression (Riboldi et al. 2019), including recent data associating the p.Gly2385Arg exonic variant with earlier onset and longer mean survival than non-carriers (Kouki et al. 2026). Associations with *GBA* variants likewise appear to vary across populations (Segarane et al. 2009; Mitsui et al. 2015). Additional loci of interest include *MAPT* subhaplotypes (Vilariño-Güell et al. 2011), which have been proposed as potential contributors to disease susceptibility, as well as repeat expansions associated with cerebellar ataxia. Diagnostic overlap with spinocerebellar ataxias is particularly likely when autonomic features, such as bladder and cardiovascular dysfunction, accompany cerebellar symptoms, potentially limiting the interpretation of these associations (Stamelou et al. 2013; Kim et al. 2014).

Such complexity is further illustrated by both the relatively high frequency of family history of parkinsonism observed in individuals with MSA (Leys et al. 2022) and by rare families in which different α-synucleinopathies co-occur. In one neuropathologically characterized pedigree including both MSA and PD with dementia, extensive genetic analyses failed to identify variants in known genes associated with parkinsonism or MSA mimics, suggesting the presence of yet unidentified genetic factors (Fanciulli et al. 2022).

More comprehensive genome-wide approaches have recently been applied to investigate the genetic basis of MSA. Integrative analyses combining genome-wide association studies (GWAS), transcriptome-wide approaches, and colocalization methods have identified candidate genes such as *USP38-DT* and *KCTD7*. Variants at these loci colocalize with cis-expression quantitative trait loci, linking genetic risk to regulatory effects on gene expression and supporting a potential role of oligodendroglial biology in MSA pathogenesis (Chia et al. 2024). A GWAS in autopsy-confirmed cases further highlighted suggestive loci, including a signal near *ZIC1/ZIC4*, with reduced ZIC4-positive neurons observed in olivopontocerebellar atrophy, supporting a role in cerebellar vulnerability (Hopfner et al. 2022). Recent X-chromosome-wide association analyses have additionally identified sex-specific susceptibility loci, including a female-associated signal at Xq28 potentially involving *FAM3A* and *PLXNA3*, and a male-associated locus at Xp22.2 within TBL1X, suggesting that sex-related genetic factors may also contribute to MSA risk (Ray et al. 2026).

The interpretation of genetic association studies in MSA, however, remains complicated by substantial diagnostic uncertainty in clinically diagnosed cases. Neuropathological examination remains the diagnostic gold standard, as no validated biomarker is currently for use in living patients, and autopsy studies have shown that up to 38% of clinically diagnosed MSA cases are misdiagnosed during life (Koga et al. 2015). This limitation likely introduces significant heterogeneity into genetic cohorts and may partly account for inconsistent findings across studies. Understanding how genetic susceptibility, within a likely polygenic architecture, interacts with the molecular mechanisms underlying MSA will therefore be essential for clarifying its contribution to disease development.

## Pathophysiological mechanisms

Neuropathologically, MSA is characterized by the abnormal accumulation of α-synuclein within oligodendrocytes, forming GCIs (Spillantini et al. 1998). Unlike the twisted, single-protofilament structures observed in PD and dementia with Lewy bodies (DLB) (Yang et al. 2022), MSA-associated aggregates consist of two distinct α-synuclein filament types, each composed of paired protofilaments that assemble into thicker, largely untwisted structures (Schweighauser et al. 2020).

An early and critical event in MSA pathogenesis is the relocalization of the myelin-associated protein p25α, also known as tubulin polymerization-promoting protein (TPPP). Under physiological conditions, p25α is confined to the myelin sheath; however, in MSA it redistributes to the oligodendroglial cell body, preceding α-synuclein aggregation. This shift promotes phosphorylation of α-synuclein at serine-129 and facilitates its aggregation into insoluble oligomers (Ferreira et al. 2021).

The mechanisms underlying α-synuclein accumulation in oligodendrocytes are not yet fully understood. While p25α overexpression enhances the recruitment of endogenous α-synuclein into pro-degenerative inclusion bodies (Song et al. 2007), the relatively low levels of *SNCA* mRNA in oligodendrocytes suggest that local overproduction alone is insufficient to account for its accumulation (Asi et al. 2014; Kon et al. 2024). Consequently, alternative mechanisms have been proposed, including the uptake of neuronal α-synuclein aggregates through the processing of damaged axonal segments (De Nuccio et al. 2023), as well as impaired clearance due to lysosomal dysfunction or altered oligodendroglial secretory pathways (Yu et al. 2020).

Experimental studies suggest that once established, α-synuclein aggregates trigger a cascade of oligodendroglial dysfunction, microglial activation, astrogliosis, and neuroinflammation (Refolo et al. 2018; Scheiblich et al. 2021; Torre-Muruzabal et al. 2023; Heras-Garvin et al. 2025; Luque et al. 2026). In parallel, progressive oligodendroglial impairment may lead to the release of misfolded α-synuclein into the extracellular space, facilitating its uptake by adjacent neurons and promoting the formation of neuronal cytoplasmic inclusions. Recent neuropathological and experimental evidence suggests that neuronal α-synuclein toxicity, rather than oligodendroglial cytoplasmic inclusions alone, may represent the principal driver of neurodegeneration in MSA. In particular, α-synuclein has been shown to accumulate early within neuronal nuclei, disrupt the nuclear envelope, and induce rapid neuronal loss, while neuronal α-synuclein aggregates appear more proteolysis-resistant and neurotoxic than the comparatively more soluble and less harmful oligodendroglial aggregates (Wiseman et al. 2025).

The neurodegenerative process is further shaped by additional factors, including dysregulated iron metabolism, mitochondrial dysfunction, impaired neurotrophic factor signaling (e.g. glial cell line-derived neurotrophic factor [GDNF]), reduced differentiation of oligodendrocyte precursor cells into mature myelinating oligodendrocytes, and altered brain insulin signaling (Stefanova and Wenning 2023; Krismer et al. 2024a), underscoring the biological heterogeneity of MSA.

## Diagnostic criteria and current challenges

A diagnosis of MSA during life still largely relies on clinical criteria and remains challenging, despite substantial advances in understanding MSA pathogenesis. Clinicopathological validation studies of the 2008 s consensus criteria (Gilman et al. 2008) have shown only moderate diagnostic accuracy, with positive predictive values ranging from approximately 62% to 79% (Miki et al. 2019), reflecting frequent misclassification with other neurodegenerative disorders, particularly PD, DLB, and progressive supranuclear palsy (PSP) (Koga et al. 2015).

In response to these limitations, the 2022 diagnostic criteria endorsed by the Movement Disorder Society introduced graded levels of diagnostic certainty (neuropathologically established, clinically established, clinically probable, and possible prodromal MSA) with the aim of improving diagnostic precision across different stages of the disease (Wenning et al. 2022). Pathological validation studies indicate that these updated criteria improve diagnostic performance, particularly by increasing specificity through the integration of characteristic clinical features and supportive imaging markers (Virameteekul et al. 2023; Sekiya et al. 2023). Structural MRI findings, including putaminal signal abnormalities, middle cerebellar peduncle atrophy, and the “hot cross bun” sign, demonstrate high specificity for MSA and are therefore incorporated into the criteria for clinically established MSA.

Their sensitivity, however, is often limited, especially in the early stages of the disease, indicating that the absence of MRI abnormalities does not exclude the diagnosis (Sekiya et al. 2023; Wilkens et al. 2024). This likely reflects the reliance on conventional neuroimaging markers. More advanced approaches, such as automated volumetric analyses, are emerging as promising tools with high diagnostic accuracy even at early disease stages, although their availability remains largely limited to specialized centres (Scherfler et al. 2016; Vaillancourt et al. 2025).

A need therefore remains for multimodal supportive biomarkers that integrate complementary approaches capturing different aspects of disease biology (Wan et al. 2023). Promising candidates include neurofilament light chain (NfL) and glial fibrillary acidic protein (GFAP), which have demonstrated good performance in distinguishing MSA from other parkinsonian disorders and healthy controls (Guo et al. 2023; Katzdobler et al. 2024; Kou et al. 2025). Nevertheless, their diagnostic specificity remains limited when considered in isolation, as elevated levels are observed across multiple neurodegenerative conditions (Parnetti et al. 2019).

To address these limitations, α-synuclein seed amplification assays (SAAs) have recently emerged as a valuable tool for distinguishing MSA from other α-synucleinopathies based on distinct fluorescence amplification patterns. Performance varies across cohorts, likely reflecting differences in experimental protocols and substrates (Grossauer et al. 2023; Ma et al. 2024), and combining SAAs with other biomarkers may better capture disease heterogeneity (Singer et al. 2020).

Diagnostic assessment can be further refined through molecular imaging. Some PET tracers show preferential binding patterns compatible with MSA compared with other α-synucleinopathies (Koga et al. 2017; Smith et al. 2023). The α-synuclein PET tracer [18 F]SPAL-T-06 demonstrated high-contrast in vivo detection of α-synuclein-pathology in patients with both the parkinsonian and cerebellar subtypes of MSA, showing increased retention in the putamen, pons and cerebellar white matter and peduncles in accordance with the distribution of GCI (Matsuoka et al. 2022). By contrast, the tracer [18 F]C05-05 successfully detected α-synuclein inclusions in the midbrain of patients with PD and DLB, whereas in MSA increased signals were observed in the basal ganglia and deep cerebellar structures specifically in patients with the parkinsonian subtype (Endo et al. 2024).

The development of such multimodal biomarkers is particularly relevant for detecting the disease at its earliest stages and has stimulated growing interest in the prodromal phase of MSA.

## Prodromal phase and early biomarkers

Increasing evidence suggests that MSA is preceded by a prodromal phase in which neurodegenerative processes are already ongoing before the full clinical syndrome becomes manifest (Xia and Postuma 2020; Osaki et al. 2021; Jecmenica-Lukic et al. 2021). During this stage, clinical features shared across prodromal α-synucleinopathies may help inform the underlying disease process. For example, REM sleep behaviour disorder (RBD) and subtle motor signs may occur both in premotor PD or DLB and in prodromal MSA, reflecting early involvement of the central nervous system (Miglis et al. 2021; Delva et al. 2025).

Consistent with this, pure autonomic failure (PAF) has emerged as a particularly informative clinical model for investigating the mechanisms underlying phenoconversion towards central α-synucleinopathies. Although PAF was historically considered an α-synucleinopathy restricted to the autonomic nervous system (Mendoza-Velásquez et al. 2019; González-Duarte et al. 2021), longitudinal studies have demonstrated that a subset of patients subsequently develops a central α-synucleinopathy (Kaufmann et al. 2017; Singer et al. 2017; Giannini et al. 2018; Coon et al. 2020; Millar Vernetti et al. 2024; Chiaro et al. 2026). A recent meta-analysis including 900 individuals with PAF showed that, over a mean follow-up of about six years, 30% of patients phenoconverted to a central α-synucleinopathy, corresponding to a pooled incidence rate of 5.09 per 100 person-years. Conversion to MSA occurred in 12% of cases and showed the highest phenoconversion rates during the early years of follow-up. The study also identified specific clinical predictors: subtle motor signs were associated with near-term phenoconversion to any α-synucleinopathy, whereas hyposmia was linked to neuronal types (PD or DLB) (Virameteekul et al. 2026). These findings are consistent with those of a recent multicentre longitudinal study, which reported relatively early phenoconversion to MSA compared with other α-synucleinopathies, suggesting that distinct disease mechanisms may already be present during the prodromal stage (Millar Vernetti et al. 2024).

Clinical predictors alone, however, may have limited diagnostic accuracy in identifying which patients with PAF will eventually develop MSA. For this reason, increasing attention has been directed towards biofluid biomarkers, such as NfL and α-synuclein seeding assays, which have shown promising potential in predicting phenoconversion to MSA during the prodromal stage (Singer et al. 2021). While these biomarkers may help identify individuals at risk of developing MSA, additional tools are needed to monitor disease progression once it becomes clinically manifest.

## Markers of disease progression

The evaluation of disease progression in MSA, as well as the assessment of response to disease-modifying therapies, has largely relied on clinical measures, most commonly the Unified MSA Rating Scale (UMSARS) (Wenning et al. 2004). While certain limitations have been identified, including variable sensitivity to longitudinal change across items, with motor features generally showing greater responsiveness than autonomic domains, the UMSARS remains a widely used instrument for assessing symptom burden and its impact on activities of daily living (Palma et al. 2021; Foubert-Samier et al. 2022; Krismer et al. 2022). Clinical scales may, however, not fully capture subtle biological changes or treatment-related effects at the individual level, prompting increasing interest in integrating objective biomarkers to complement clinical outcome measures, with neuroimaging emerging as a particularly informative approach.

Longitudinal MRI studies have shown significant brain volume loss in MSA over relatively short timeframes, particularly affecting the cerebellum, pons, and putamen (Krismer et al. 2024b). In addition, several MRI markers have been shown to correlate with disease subtype and symptom severity, particularly when imaging findings are integrated with clinical or metabolic data (Jin et al. 2025). Beyond structural MRI, more advanced functional and molecular imaging techniques may further refine the characterization of disease progression (Krismer et al. 2025).

Consistent with these findings, the ASPIRE study applied a multimodal approach combining clinical measures with MRI, DaT-SPECT imaging, and plasma biomarkers in patients with early MSA (Fabbri et al. 2026). Measurable neuroimaging changes were detectable within six months, reflecting the aggressive course of the disease, with cerebellar and pontine atrophy predominating in MSA-C and progressive reductions in striatal dopaminergic binding in MSA-P. Baseline brainstem and pontine atrophy predicted clinical worsening over a six-month period, whereas baseline DaT-SPECT measures showed stronger predictive value over 12 months. Higher baseline plasma NfL levels were also associated with faster clinical progression, greater disability, and increased mortality, highlighting its value as a prognostic biomarker.

A multimodal biomarker approach to disease progression may be particularly useful for optimizing patient selection and outcome measures in clinical trials, with potential implications for the development of disease-modifying therapies.

## Disease-modifying therapies

Advances in biomarker research and in the understanding of disease mechanisms, together with regulatory incentives such as orphan disease designation, have stimulated increasing efforts to develop disease-modifying therapies for MSA.

Most therapeutic strategies have focused on different stages of the α-synuclein pathway, with a range of pharmacological and immunotherapeutic approaches developed to target these processes (Sidoroff et al. 2022). They include *SNCA*-targeting strategies such as intrathecally administered antisense oligonucleotides (ASOs) designed to reduce *SNCA* mRNA and α-synuclein protein production [NCT04165486], small molecules designed to inhibit aggregation or modulate oligomer formation, active and passive immunotherapies aimed at limiting the spread of pathological α-synuclein, and compounds intended to enhance degradation pathways such as the autophagy-lysosomal system (Table 1) (Krismer et al. 2024a; Liu et al. 2024).

**Table 1:** Ongoing therapeutic trials targeting the α-synuclein pathway in MSA

Stage of α-synuclein cascade	Therapeutic approach	Representative agents	Clinical development stage	Key outcomes
Production	Gene silencing (ASOs)	ION464	Phase I	Targets *SNCA* mRNA to reduce α-synuclein synthesis [Ionis. Pipeline]
Misfolding/oligomerization	Oligomer modulation (small molecules)	TEV-56,286 (Emrusolmin)	Phase II	Modulates toxic oligomer formation [NCT06568237]
	c-Abl inhibition	IkT-148,009	Phase I	Reduces α-synuclein phosphorylation/aggregation [Inhibikase Therapeutics. Press Release]
Aggregation/oxidative stress	Iron-modulating small molecule	ATH434	Phase II	Reduced brain accumulation and showed slower clinical progression over 52 weeks (Alterity Therapeutics 2026)
Propagation	Passive immunotherapy	Lu AF82422 (Amlenetug)	Phase III	Targets extracellular α-synuclein species to limit cell-to-cell spread [NCT06706622]
	Passive immunotherapy	TAK-341	Phase II	Monoclonal antibody against α-synuclein [P9-9.003]
	Active immunotherapy	PD01/PD03A	Phase I	Induces endogenous anti-α-synuclein antibodies (Meissner et al. 2020)
	Active immunotherapy	UB-312	Phase I	Peptide-based vaccine targeting α-synuclein (Eijsvogel et al. 2024)

*ASO* antisense oligonucleotide, *SNCA* gene encoding α-synuclein

Several a-synuclein targeting interventions have shown promising effects in experimental models (Kallab et al. 2018; Heras-Garvin et al. 2019, 2021; Herrera-Vaquero et al. 2019; Lemos et al. 2020). Multiple clinical trials applying immunotherapies are ongoing [NCT06706622; NCT05104476; NCT05634876; NCT06015841]. In particular, the MASCOT and AMULET studies investigate amlenetug (Lu AF82422), a humanized monoclonal antibody targeting extracellular aggregated α-synuclein with the aim of preventing pathological seeding and cell-to-cell propagation. Although the Phase 2 AMULET trial did not meet its primary endpoint, amlenetug showed an acceptable safety profile and a non-significant trend toward slowing clinical progression, supporting further evaluation in Phase 3 studies (Kjærsgaard et al. 2026). In parallel, clinical trials are focusing on small molecules that target a-synuclein aggregation. One example is TEV-56,286 (experimentally known as Anle138b), which is currently being tested in participants with MSA [NCT06568237], following encouraging preclinical findings (Heras-Garvin et al. 2019). Another promising candidate is the iron-modulating small molecule ATH434. Consistent with the preclinical evidence (Heras-Garvin et al. 2021; Finkelstein et al. 2022), ATH434 has shown reductions in brain iron accumulation and slower clinical progression over 52 weeks, with a safety profile comparable to placebo (Alterity Therapeutics 2026).

Targeting pathological α-synuclein, however, may be insufficient to alter disease progression, highlighting the need to move beyond protein clearance towards strategies that also restore physiological protein function. Additional pathogenic mechanisms, including neuroinflammation, have been explored as therapeutic targets. Early studies with agents such as minocycline and the myeloperoxidase inhibitor verdiperstat yielded controversial results in different experimental settings dependent on the time of initiation of the therapy (Stefanova et al. 2004, 2007, 2012; Kaindlstorfer et al. 2015). These findings were reflected by the lack of significant clinical benefit in later trials despite the imaging evidence of target engagement (Dodel et al. 2010).

Recent experimental studies have focused on targeting the NLRP3-inflammasome in MSA and have demonstrated a very strong rationale for the strategy, including both preventive and therapeutic efficacy after the application of clinically safe drug dosing, mechanistic relevance to human disease-associated microglia signatures, and a set of putative fluid biomarkers for monitoring of treatment effects (Luque et al. 2026). Further agents like S1P5 receptor modulators [NCT05923866] and compounds modulating inflammasome-related signaling (Tseng 2024; NCT06848231), are currently under clinical investigation.

Metabolic approaches targeting mitochondrial dysfunction have also been explored, and recent studies have associated high-dose ubiquinol supplementation with a smaller decline in motor scores (Mitsui et al. 2023). On the other hand, other putative neuroprotective agents, including riluzole and rasagiline, have failed to demonstrate convincing clinical efficacy (Seppi et al. 2006; Bensimon et al. 2009).

Recent disease-modifying strategies have increasingly focused on restoring neuronal integrity. Cell-based therapies using autologous mesenchymal stem cells from bone marrow or adipose tissue have shown potential to modulate neuroinflammation and provide neurotrophic support, with early clinical studies suggesting a possible slowing of functional decline and preservation of gray matter density (Lee et al. 2012; Singer et al. 2019; Chung et al. 2021). Larger randomized controlled trials are required to confirm safety and efficacy.

A complementary approach uses gene therapy to restore neurotrophic signalling within vulnerable neuronal circuits. Viral vector–mediated delivery of glial cell line–derived neurotrophic factor (GDNF), particularly the AB-1005 platform [P2-3.004], is designed to enhance neuronal survival and resilience rather than directly targeting protein aggregation. Although still preliminary, restoration of neuronal function is emerging as a potential research direction in α-synucleinopathies, together with strategies aimed at reducing the burden of pathogenic molecular alterations.

## Clinical management

Current management of MSA remains primarily symptomatic and requires a comprehensive, multidisciplinary approach focused on preserving functional autonomy (Fanciulli and Wenning 2015). Achieving these goals requires coordinated care from neurologists, physiotherapists, speech and language therapists, autonomic specialists, and palliative care teams, reflecting the complex combination of motor and non-motor manifestations that frequently coexist and interact in this disorder (Sorrentino et al. 2026).

Motor symptoms generally show a limited or transient response to dopaminergic therapy compared with PD, making non-pharmacological strategies particularly important. Targeted physiotherapy and occupational therapy play a key role in maintaining mobility, addressing gait impairment, and reducing the high risk of falls associated with the disease (Krismer et al. 2024a).

Progressive bulbar and respiratory dysfunction further complicates the clinical management of MSA. Dysphagia may contribute to malnutrition, dehydration, and aspiration pneumonia, making regular swallowing assessment and nutritional support essential, while percutaneous endoscopic gastrostomy may need to be considered in advanced stages (Brown et al. 2020; Ruttle et al. 2026). Laryngeal dysfunction, including vocal cord paralysis and stridor, can lead to respiratory complications and an increased risk of sudden death, occasionally requiring interventions such as non-invasive ventilation or tracheostomy (Gandor et al. 2020). As the complications often emerge during disease progression, decisions regarding gastrostomy and tracheostomy should be addressed within a multidisciplinary framework and aligned with the preferences and goals of care of patients and their families.

Autonomic dysfunction is another core feature and a major therapeutic priority. Orthostatic hypotension, in particular, can be highly disabling and often requires a combination of lifestyle measures and pharmacologic treatment (Fanciulli et al. 2026). Stabilizing blood pressure is essential not only to prevent syncope and injury but also to improve fatigue, cognitive fluctuations, and symptoms of orthostatic intolerance (Calió et al. 2025).

Both motor and autonomic disturbances contribute not only to disability but also to a broader spectrum of non-motor symptoms, including pain, which is increasingly recognized as a significant clinical component of MSA. The characteristic “coat-hanger” pain is one example, but it captures only part of a broader clinical spectrum. In a recent community-based survey, up to 87% of individuals with MSA reported experiencing pain, highlighting its relevance as a clinical feature throughout the disease course (Campese et al. 2024). These observations suggest that pain in MSA is not only common but also multifactorial, reflecting the interplay of different pathological mechanisms, including rigidity, dystonia, postural abnormalities, autonomic dysfunction, and comorbid conditions. Recognizing this multidimensional nature is important in clinical practice, as pain may be underestimated or attributed to a single mechanism, while its management often requires a more comprehensive and individualized approach (Campese et al. 2026). In this context, neuropathic mechanisms appear to play a less prominent role, and the frequent use of analgesics may therefore reflect a therapeutic gap, as symptoms often improve when the underlying motor or autonomic dysfunction is more effectively addressed (Campese et al. 2023).

Sex-related differences in the clinical expression of MSA may also influence management strategies. Evidence suggests that men more often develop severe cardiovascular autonomic dysfunction, including earlier and more pronounced orthostatic hypotension, whereas women may present with greater motor disability and a higher neuropsychiatric burden, particularly depression (Leys et al. 2024). Recognizing these differences is important for tailoring both pharmacological interventions and multidisciplinary support to each patient’s specific needs.

## Healthcare provision in MSA: challenges and arising models

Maintaining regular follow-ups in clinical practice, however, remains challenging. As the disease progresses and mobility declines, many patients experience increasing difficulty attending visits at tertiary referral centres and may gradually lose access to specialized care. These barriers became particularly evident during the COVID-19 pandemic, when restrictions and reduced hospital access.

further interrupted established care pathways. Temporary closures of specialized centres and interruptions in rehabilitation services disrupted both diagnostic processes and routine follow-up, highlighting how strongly many patients depend on in-person support (Fanciulli et al. 2023a). At the same time, the rapid expansion of digital health technologies revealed new opportunities for care delivery. Telemedicine and hybrid models may help reduce geographical and mobility barriers, allowing clinicians to monitor symptoms longitudinally while observing aspects of daily functioning within the patient’s home environment. For individuals with progressive autonomic disorders, such approaches may provide an important bridge between specialized centres and the realities of everyday life.

Early studies using these approaches adopted hybrid models that combined in-person visits with remote interactions, including programs focused on therapeutic education for patients and caregivers and nurse-led remote monitoring to enable early detection of complications and improve care coordination (Cilia et al. 2025; Fabbri et al. 2025). In this context, the Medical Decision Making in Multiple System Atrophy (MeDeMSA) Care study, an ongoing 18-month monocentric study, is evaluating whether a patient-centred, multidisciplinary model that integrates telemedicine with individualized treatment plans, including pharmacological and non-pharmacological interventions, can improve quality of life in individuals with MSA (Fanciulli et al. 2025). These initiatives suggest that new models may improve access to specialized care in MSA, although their impact on patients’ quality of life remains to be clarified.

## Discussion

MSA remains a highly challenging neurodegenerative disorder from both diagnostic and therapeutic perspectives. Despite important advances in understanding its molecular mechanisms, genetic background, and biomarker development, early diagnosis remains difficult, and disease-modifying therapies are still lacking. This gap between biological insight and clinical impact is one of the central challenges in the field. As a result, improving outcomes for individuals with MSA requires not only continued scientific progress but also the development of clinical care models that translate these advances into timely diagnosis, coordinated management, and sustained support throughout the disease course.

One major barrier to early recognition is the unequal availability of expertise in autonomic medicine. Although the number of autonomic laboratories in Europe has increased in recent years, their distribution remains highly heterogeneous, with many services concentrated in Northern and Western countries (Habek et al. 2022). Clinical expertise also varies substantially across centres, and many neurologists report limited confidence in managing hallmark autonomic manifestations, such as bladder, cardiovascular, or sexual dysfunction. Consequently, patients with MSA are often evaluated in clinical settings where autonomic symptoms may be underrecognized, despite their diagnostic relevance and significant impact on quality of life (Reis-Carneiro et al. 2024). Strengthening networks of specialized autonomic centres and improving training in autonomic disorders may therefore be important steps towards improving diagnostic accuracy and facilitating access to appropriate care (Fanciulli et al. 2023b).

Beyond diagnostic challenges, the moment of diagnosis is a critical turning point in the disease trajectory. For clinicians, the focus is often on symptomatic treatment and clinical monitoring. For patients and families, however, receiving an MSA diagnosis frequently triggers profound uncertainty about independence, daily functioning, and future decline. At this stage, a clear clinical roadmap is essential. Early, transparent communication, anticipatory guidance, and timely discussions of future needs and wishes should be considered fundamental components of clinical management. Advance care planning, including goals of care, resuscitation preferences, and end-of-life decisions, should be initiated early in the disease course, well before cognitive or communication impairments limit the patient’s ability to express their preferences (Breitegger et al. 2024). Together, these approaches can help reduce uncertainty, support shared decision-making, and enable patients and caregivers to prepare for the disease’s progressive course (Kluger 2025).

Patients and caregivers, consequently, become central actors in interpreting symptoms and responding to daily challenges. Although limited professional guidance may sometimes lead to suboptimal strategies, patients often develop practical ways of adapting to symptoms within their own environment (Caliò et al. 2026). These experiences offer valuable insight into the realities of living with MSA and frequently reveal needs that are difficult to capture during routine clinical controls.

**Fig. 1:**
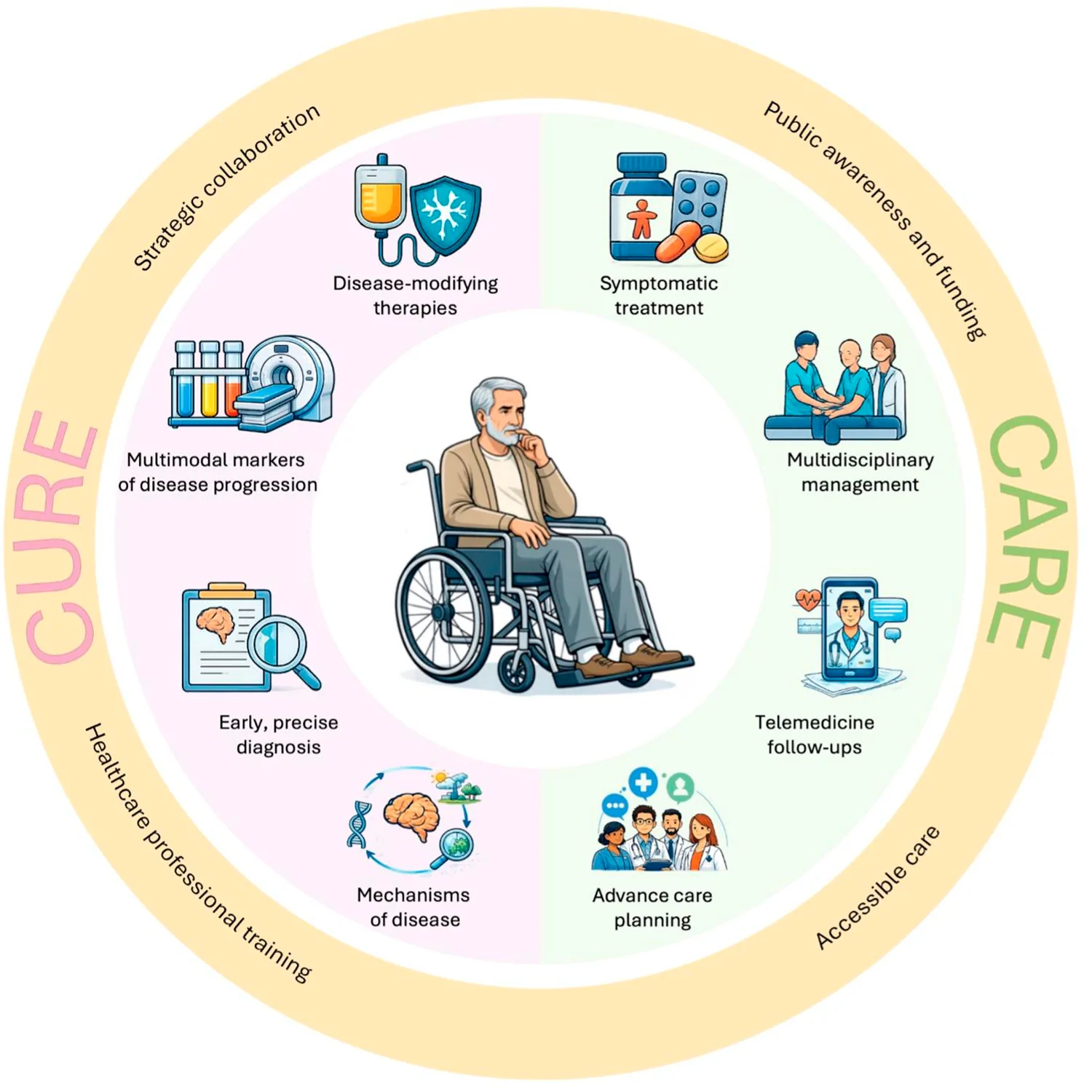
The figure illustrates the dual framework of curing and caring for people living with MSA. On the cure side, advances in understanding the mechanisms of disease, biomarkers and diagnostic approaches support earlier and more precise disease identification, enabling the development of disease-modifying therapies. On the care side, management relies on symptomatic treatment, multidisciplinary approaches, telemedicine follow-ups and advance care planning aimed at improving quality of life throughout the disease course. These domains are embedded within a broader healthcare context that includes healthcare professional training, public awareness and dedicated research funding, accessible care, and strategic collaboration, all of which are essential to translate scientific advances into effective clinical practice

Improving outcomes for individuals with MSA will require integrated models that bridge advances in understanding disease mechanisms and therapeutic development with comprehensive care strategies, including symptomatic treatment, multidisciplinary collaboration, remote monitoring, and advance care planning across the entire disease trajectory (Fig. 1). Such models should not only address the biological complexity of the disorder but also place patient centricity at the core of clinical management, recognizing patients and caregivers as active partners in both the cure and care process.

Generative AI (OpenAI) was used to design figures and convert graphical intellectual content of the authors into text.

## Data Availability

No datasets were generated or analysed during the current study.
